# The impact of bariatric surgery on serum tryptophan–kynurenine pathway metabolites

**DOI:** 10.1038/s41598-021-03833-4

**Published:** 2022-01-07

**Authors:** Kai Tai Derek Yeung, Nicholas Penney, Luke Whiley, Hutan Ashrafian, Matthew R. Lewis, Sanjay Purkayastha, Ara Darzi, Elaine Holmes

**Affiliations:** 1 Department of Surgery & Cancer, Imperial College London, South Kensington, London, UK; 2 Australian National Phenome Centre & Centre for Computational & Systems Medicine, Health Futures Institute, Murdoch University, Perth, WA Australia; 3 National Phenome Centre, Imperial College London, South Kensington, London, UK; 4 Department of Metabolism, Digestion and Reproduction, Imperial College London, South Kensington, London, UK

**Keywords:** Metabolomics, Endocrine system and metabolic diseases, Metabolic disorders

## Abstract

This study aims to explore the immediate effects of bariatric surgery on serum tryptophan–kynurenine pathway metabolites in individuals with type 2 diabetes and BMI > 30. With the goal of providing insight into the link between tryptophan pathway metabolites, type 2 diabetes, and chronic obesity-induced inflammation. This longitudinal study included 20 participants. Half were diagnosed with type 2 diabetes. 11 and 9 underwent RYGB and SG respectively. Blood samples were obtained at pre-operative and 3 months post-operative timepoints. Tryptophan and downstream metabolites of the kynurenine pathway were quantified with an ultrahigh-performance liquid chromatography tandem mass spectrometry with electrospray ionisation method. At 3 months post-operation, RYGB led to significant reductions in tryptophan, kynurenic acid and xanthurenic acid levels when compared to baseline. Significant reductions of the same metabolites after surgery were also observed in individuals with T2D irrespective of surgical procedure. These metabolites were significantly correlated with serum HbA1c levels and BMI. Bariatric surgery, in particular RYGB reduces serum levels of tryptophan and its downstream kynurenine metabolites. These metabolites are associated with T2D and thought to be potentially mechanistic in the systemic processes of obesity induced inflammation leading to insulin resistance. Its reduction after surgery is associated with an improvement in glycaemic control (HbA1c).

## Introduction

Morbid obesity is a growing global epidemic associated with a number of significant health conditions leading to a chronic and increasingly heavy burden on healthcare systems. Co-morbidities include type 2 diabetes (T2D), hypertension, cardiovascular disease, non-alcoholic fatty liver disease, chronic kidney disease, certain cancers and infertility.

Bariatric surgery is one of the most effective long-term treatments for patients with morbid obesity and its associated co-morbidities^1–3^. It leads to significantly improved glycaemic control^1,4^, and reduced long term incidences of T2D-associated complications^5^. There are many postulated mechanisms of action, which contribute to such improvements, including calorie restriction, weight loss, modifications in bile acid metabolism, alteration in gut hormones and in the gut microbiome^6^. Some of these changes seem to occur in the early post-operative period and may be independent to weight loss^7^. The different types of bariatric surgery have different success rates with respect to weight loss, improvement of cardiovascular disease risk and remission of sleep apnoea with the Roux-En-Y gastric bypass (RYGB) achieving the most noteworthy results^8,9^. There is also currently conflicting evidence and debate as to whether certain types of operations favour T2D remission^8,10–12^.

Patients with obesity have an underlying chronic low-grade systemic inflammatory state. Pro-inflammatory factors are secreted by immune cells that accumulate in visceral adipose tissue^13^. Previously it has been reported that this systemic inflammation is reflected in an individual’s metabolic phenotype^14,15^ with metabolites from the tryptophan (TRP)–kynurenine (KYN) pathway thought to be potentially mechanistic in the systemic processes that lead to clinical insulin resistance^15^.

**Figure 1:**
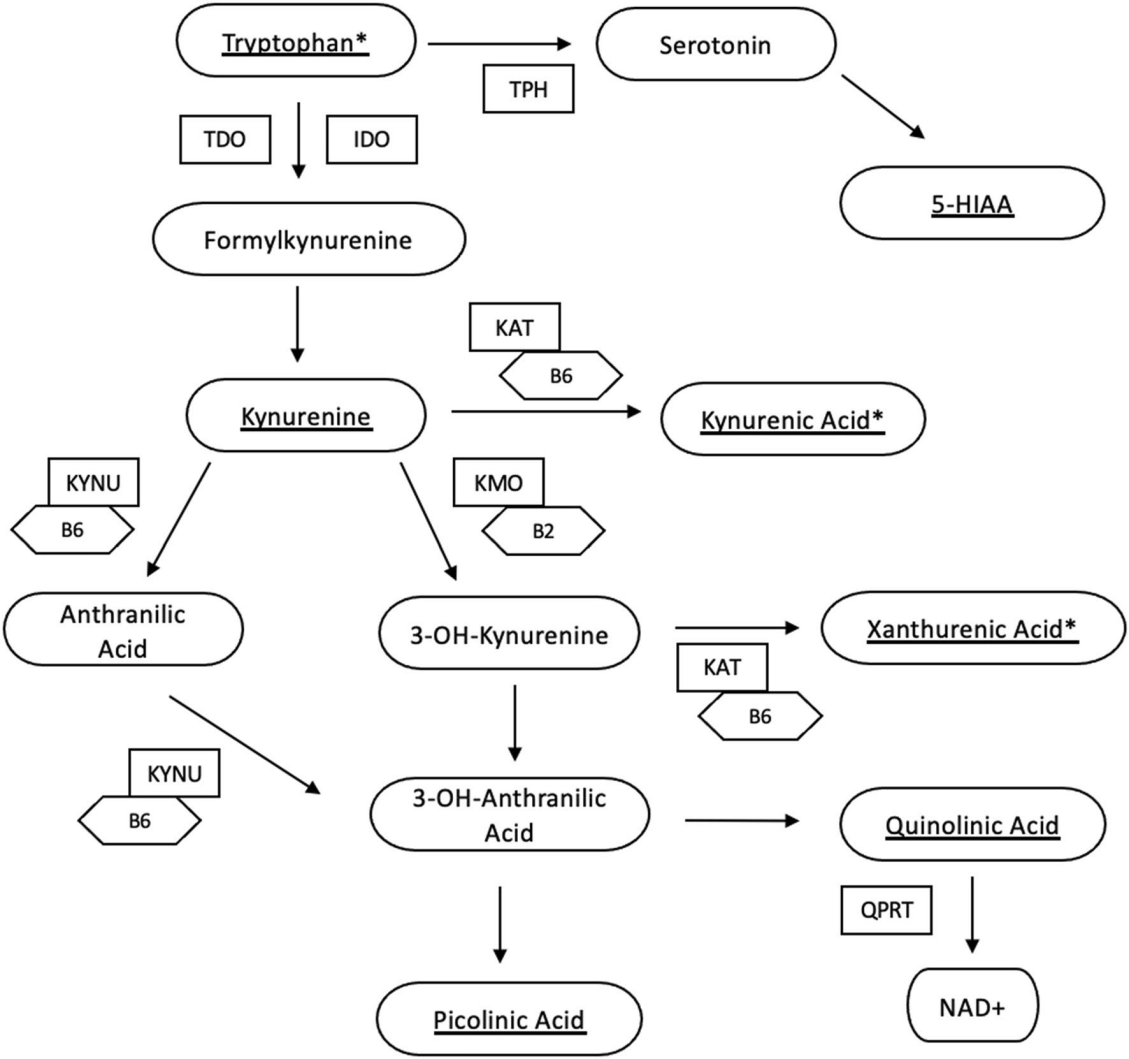
Tryptophan metabolism via kynurenine and serotonin metabolic routes. Underlined metabolites were quantified in this study. *Indicates key metabolites discussed in this study. Key: *TPH* tryptophan hydroxylase, *TDO* tryptophan 2,3-dioxygenase, *IDO* indoleamine 2,3-dioxygenase, *KAT* kynurenine aminotransaminase, *KMO* kynurenine 3 monooxygenase, *KYNU* kynureninase, *Vit B6* pyridoxal 5-phosphate, *Vit B2* Flavin Adenine Dinocleotide, *5-HIAA* 5-Hydroxyindoleacetic acid, *NAD* nicotinamide adenine dinucleotide, *QPRT* quinolinate phosphoribosyltransferase.

TRP is an essential amino acid found in food sources such as milk, meat, fish, eggs and cocoa. Whilst TRP is critical for endogenous protein synthesis^16^, it is also catabolised into bioactive metabolites including kynurenines, serotonin and indoles, which are components of pathways that have a wide range of systemic effects on the gut, brain, nervous system and microbiome as well as the regulation of systemic inflammation^17^. Most of the bioavailable tryptophan is oxidised and subsequently catabolised via the kynurenine pathway (Fig. 1). Further minor routes of catabolism include hydroxylation (serotonin), decarboxylation (tryptamine), and transamination (indolepyruvic acid)^18^.

The TRP–KYN pathway is of particular interest in obesity and has associations with the systemic inflammation that occurs in the condition. Driving this association is the first step in the pathway, TRP to KYN, which is catalysed by the enzymes tryptophan 2,3 dioxygenase (TDO) in the liver, or by indoleamine 2,3-dioxygenase (IDO) in extrahepatic tissues. IDO is preferentially induced by inflammatory cytokines such the Th1-type cytokine IFN-γ^19^. In obesity, such cytokines are produced by pro-inflammatory macrophages found within adipose tissue^13^. Furthermore, the kynurenine tryptophan ratio (KTR) is used as a measure of IDO activation and, by extension, cellular immune activation^20^. A higher KTR is associated with insulin resistance and reported to be indicative of the resultant systemic inflammation^21^.

Further linking the pathway to obesity, KYN can then be hydroxylated to 3-hydroxy-l-kynurenine (3OH-KYN) by kynurenine 3-monooxygenase (KMO), which is present in macrophages within adipose tissue but not primary adipocytes. 3OH-KYN is subsequently metabolised to either, xanthurenic acid (XA), or 3 hydroxyanthranilic acid. A shift towards overproduction of XA within this pathway has been associated with the impairment of production, release and the consequent  metabolic effects of insulin^21^.

In summary, obesity leads to chronic systemic inflammation. Metabolites from the TRP-KYN pathway are potentially mechanistic in this process as well as in the development of obesity related co-morbidities such as T2D.

Bariatric surgery leads to rapid and sustained resolution of obesity and its related co-morbidities. This study aims to utilise these powerful metabolic effects to provide an insight into the link between tryptophan–kynurenine pathway metabolites, T2D, and obesity induced systemic inflammation. Its implications include identifying mechanisms of action that lead to such beneficial metabolic benefits and potentially the identification of future surgical or pharmalogical therapeutic targets.

## Study design and methods

### Sample procurement and spectrometric analysis

Study participants were recruited prospectively between July 2015 to December 2016 and fulfilled criteria for bariatric surgery in line with National Institute for Health and Care Excellence guidance^22^. All individuals underwent local multidisciplinary team review and approval prior to proceeding to undergo Roux-En-Y Gastric Bypass (RYGB) or Vertical Sleeve Gastrectomy (VSG) surgery at St Mary’s Hospital, London, United Kingdom between August 2015 and July 2018. Specific inclusion and exclusion criteria are listed in Supplement 1.

Clinical parameters were measured, and blood samples were drawn as part of the routine pre- and 3-month post-operative outpatient clinical review appointments. Blood was collected using additive free red top BD Vacutainer® (New Jersey, USA) tubes. After 45 min at room temperature, the tubes were centrifuged at 12,000×*g* for 15 min. The resultant serum supernatant was aliquoted and stored at − 80 °C until analysis.

TRP and the downstream metabolites of the KYN pathway were quantified for all samples and a series of pooled QC samples, using an established targeted ultrahigh-performance liquid chromatography tandem mass spectrometry method utilising electrospray ionisation (UHPLC-ESI–MS/MS)^23^. In addition to direct TRP metabolites, neopterin was also quantified as a marker of cellular inflammation as it is produced by IFN-γ stimulated macrophages. Citrulline was measured as a marker of gut health related to enterocyte mass reduction^24^. Indole-3-acetic acid (IAA) is a gut microbiota-derived metabolite from dietary TRP^25^ and was measured as a marker of microbiome influence on the indole pathway.

### Statistical analysis

Following acquisition, mass spectrometry data were integrated using an established protocol described by Whiley et al. that employed quantification calibration and appropriate quality control checks^23^ which resulted in a concentration value for each metabolite. Concentration data points that were outside of three standard deviations from the mean were considered outliers and excluded from analysis.

The Shapiro Wilk test was performed on all measured metabolites to determine normality. The majority of the measured metabolites were not normally distributed (see Supplement 2). Significant differences in tryptophan pathway metabolite abundances between surgical intervention and diabetes subgroups were established using the non-parametric Mann–Whitney U test. To control for false discovery rate (FDR), q values were generated using the method described by Benjamini and Hochberg (BH)^26^. A q value FDR threshold of < 0.05 was employed throughout the study. Repeated measures correlation (rmcorr)^27–29^ was employed to measure the common regression slope of paired measures for multiple individuals. The calculated repeated measures coefficient (r_rm_) is interpreted in the same manner as a correlation coefficient with a value of 1 suggested of data with perfect paired association and a value of − 1 suggestive of a perfect negative association. The difference Δ (3 months—baseline) of measured metabolites was also evaluated for each participant to further investigate the effect of bariatric surgery on these metabolites. Correlations between variables was assessed using Spearman’s Rank correlation. All statistical analysis and graphical representations were conducted and produced with R (v 4.0.1) run in R Studio (v1.3.959).

### Study approval and informed consent

The study was approved by East of Scotland Research Ethics Committee (15/ES/0026). Joint Research Compliance Reference Number 15SM2479 and IRAS project ID 169767.

All procedures performed in studies involving human participants were in accordance with the ethical standards of the institutional and/or national research committee and with the 1964 Helsinki declaration and its later amendments or comparable ethical standards. Informed consent was obtained from all individual participants included in the study.

## Results

### Study baseline characteristics

Of the 20 patients included in the study (mean age and BMI of 47.05 and 49 respectively), 11 and 9 patients underwent RYGB and SG respectively. Half the patients had T2D. The overall baseline demographics prior to subgroup division are displayed in Table 1.

**Table.1:** Baseline demographics.

**Baseline demographics**	
n	20
Sex, n (M:F)	20 (5:15)
Age (years)	47.05 ± 9.23
Weight (kg)	131.00 ± 21.34
BMI (kg/m^2^)	49.00 ± 7.48
T2D, n (%)	10 (50)
HbAlc (mmols/mol)	51.35 ± 18.18
Metformin, n (%)	9 (45)
Insulin, n (%)	2 (5)

All listed data are means ± standard deviation unless stated otherwise.

The study cohort was then divided into the following four subgroups: individuals with and without T2D prior to surgery, and those stratified by their surgical intervention (RYGB or VSG) (Tables 2, 3). Separate sets of analysis were completed to compare the impact of bariatric surgery on the TRP-KYN pathway in participants with T2D vs Non-Diabetes (ND) and RYGB vs VSG.

**Table.2:** Effect of surgery by surgical procedure subgroups.

Subgroups	RYGB		VSG	
	Pre Op	Post Op	Pre Op	Post Op
n	11		9	
Sex, n (M:F)	1:10		4:5	
Age (years)	48.27 ± 9.65		45.56 ± 9.00	
Weight (kg)	124.73 ± 18.80	98.03 ± 14.04*	138.67 ± 22.78	114.80 ± 20.63*
BMI (kg/m^2^)	48.32 ± 6.12	38.03 ± 4.88*	49.81 ± 9.20	41.19 ± 7.99
T2D, n (%)	7 (64)		3 (33)	
Metformin, n (%)	6 (55)	4 (36)	3 (33)	3 (33)
Insulin, n (%)	2 (18)	1 (9)	0 (0)	0 (0)
HbA1c (mmols/mol)	51 ± 17.78	38.09 ± 7.73*	51.78 ± 19.75	39.00 ± 7.52

*p < 0.05 pre- and post-op comparison.

**Table.3:** Effect of surgery by T2D diagnosis subgroups.

Subgroups	T2D		Non diabetes	
	Pre Op	Post Op	Pre Op	Post Op
n	10		10	
Sex, n (M:F)	4:6		1:9	
Age (years)	51.7 ± 6.29		42.20 ± 9.59	
Weight (kg)	130.40 ± 22.00	104.06 ± 17.45*	131.60 ± 21.83	107.06 ± 21.02*
BMI (kg/m^2^)	48.14 ± 7.60	38.43 ± 5.97*	49.85 ± 7.66	40.48 ± 7.12*
Metformin, n (%)	9 (90)	7 (70)	0 (0)	0 (0)
Insulin, n (%)	2 (20)	1 (10)	0 (0)	0 (0)
HbA1c (mmols/mol)	65.10 ± 16.41^+^	43.20 ± 7.57*	37.60 ± 2.95	33.80 ± 3.26*
RYGB, n (%)	7 (70)		4 (40)	
VSG, n (%)	3 (30)		6 (60)	

^+^Significant difference between baseline demographics.

*p < 0.05 pre- and post-op comparison.

### Pre-operative and 3-month post-operative metabolite comparisons—surgical intervention

Both surgical interventions led to significant reductions in weight across all subgroups at 3 months post-operation (Table 2). HbA1c concentrations were significantly reduced at the 3-month timepoint following RYGB, but not within the VSG group (Table 2).

**Figure 2:**
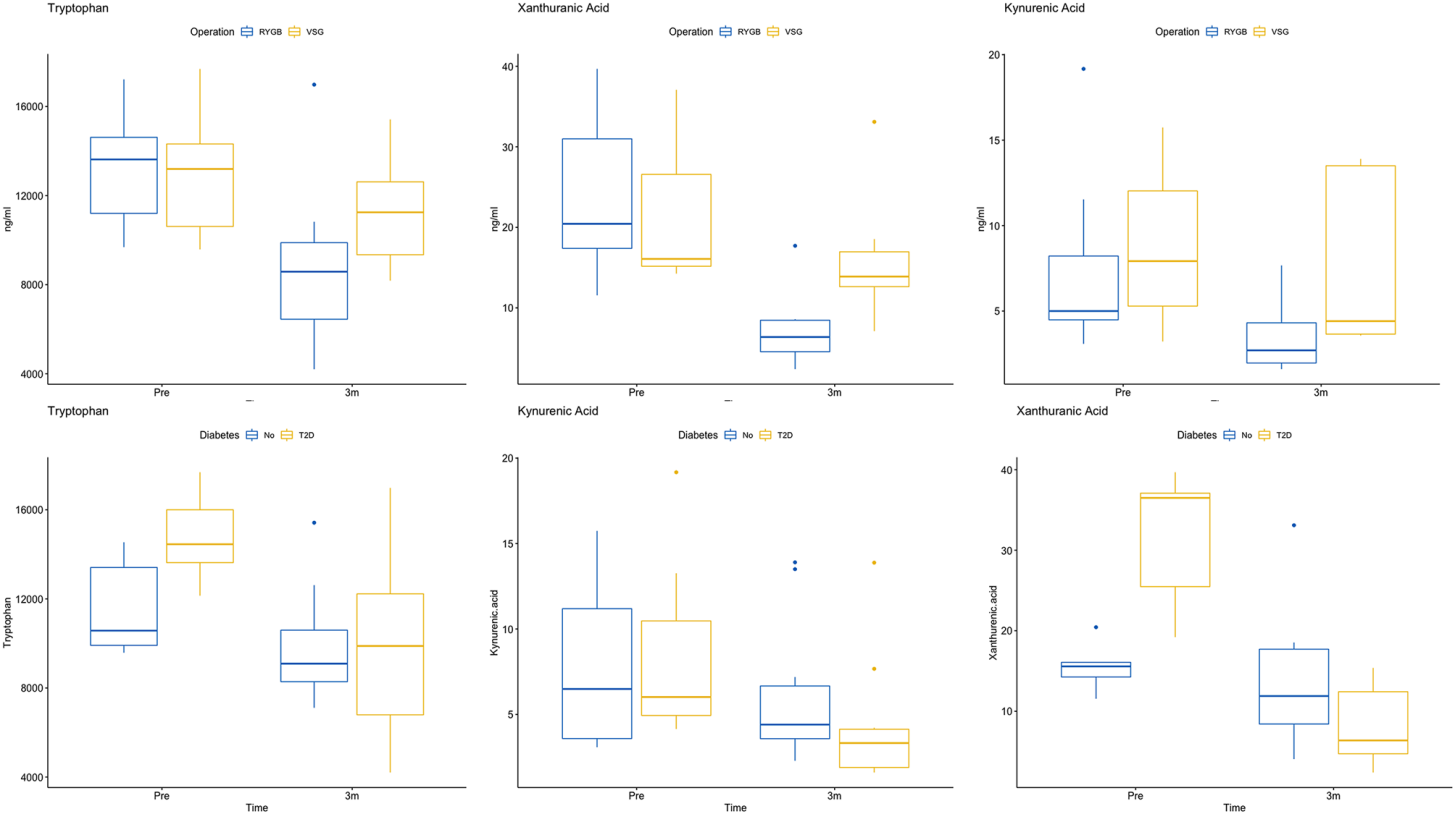
Pre- and post operative concentrations of tryptophan, kynurenic acid and xanthurenic acid shown with surgical procedure (top row) and T2D subgroups (bottom row).

RYGB intervention resulted in a significant decrease in kynurenic acid (KYNA) (p = 0.010, BH q = 0.048), XA (p = 0.001, BH q = 0.017) and TRP (p = 0.002, BH q = 0.017) concentrations (Fig. 2). Concentrations of 3-OH kynurenine (p = 0.023, BH q = 0.082) and quinolinic acid (p = 0.043, BH q = 0.121) also decreased, however presented BH q values of > 0.05 post control of the FDR. The VSG procedure did not result in any significant changes in metabolite concentrations at the 3-month timepoint (Table 4) but post-operative trend changes mirrored those observed in the RYGB group. No significant difference was found in the kynurenine/tryptophan ratio for either the RGYB or VSG groups after surgery.

**Table.4:** Effect of surgery by surgical procedure subgroup on measured metabolites.

	RYGB				VSG			
	Pre Op	Post Op	p value	q value	Pre Op	Post Op	p value	q value
Picolinic acid	7.39 ± 3.93	5.98 ± 1.51	0.933	0.933	5.32 ± 1.28	6.58 ± 1.73	0.181	0.699
3 Hydroxyanthranilic acid	15.67 ± 8.77	14.79 ± 0.59	0.533	0.747	15.31 ± 3.1	13.32 ± NA	0.857	0.923
Quinolinic acid	67.49 ± 15.35	53.95 ± 24.84	0.043	0.121	68.44 ± 20.14	64.55 ± 17.87	0.605	0.804
Citrulline	3548.68 ± 1608.63	2790.11 ± 840.02	0.217	0.434	4264.1 ± 1337.04	3296.14 ± 880.96	0.113	0.699
Indole 3 acetic acid	319.93 ± 269.57	425.13 ± 350.29	0.401	0.624	264.97 ± 154.65	361.19 ± 268.67	0.666	0.804
Kynurenic acid	7.06 ± 4.75	3.34 ± 1.87	0.010	**0.048**	8.89 ± 4.48	7.5 ± 4.83	0.596	0.804
5-Hydroxyindole acetic acid	9.53 ± 5.06	8.33 ± 2.49	0.847	0.912	8.09 ± 3.82	6.36 ± 1.49	0.340	0.794
Xanthurenic acid	24.06 ± 10.53	7.33 ± 4.78	0.001	**0.017**	22.47 ± 12.68	16.19 ± 8.23	0.267	0.747
Kynurenine	317.55 ± 69.72	248.94 ± 77.3	0.065	0.152	317.64 ± 73.11	297.82 ± 65.44	0.606	0.804
30H Kynurenine	10.56 ± 4.35	6.81 ± 2.65	0.023	0.082	10.13 ± 3.13	7.35 ± 2.03	0.063	0.699
Neopterin	2.67 ± 1.59	2.91 ± 1.93	0.844	0.912	2.35 ± 0.94	2.44 ± 0.65	0.489	0.804
Tryptophan	13,242.5 ± 2443.76	8711.05 ± 3482.81	0.002	**0.017**	12,892.19 ± 2741.4	11,291.87 ± 2413.88	0.200	0.699
Kynurenine/tryptophan ratio	0.025 ± 0.006	0.026 ± 0.007	0.842	0.912	0.024 ± 0.004	0.026 ± 0.004	0.689	0.804

q values were generated using the Benjamini and Hochberg method to control the false discovery rate (FDR).

Units of all of metabolites displayed—ng/ml.

KTR expressed as a ratio.

Significant values are indicated by bold type.

### Pre-operative and 3-month post-operative metabolite comparisons—T2D vs non-diabetes

**Figure 3:**
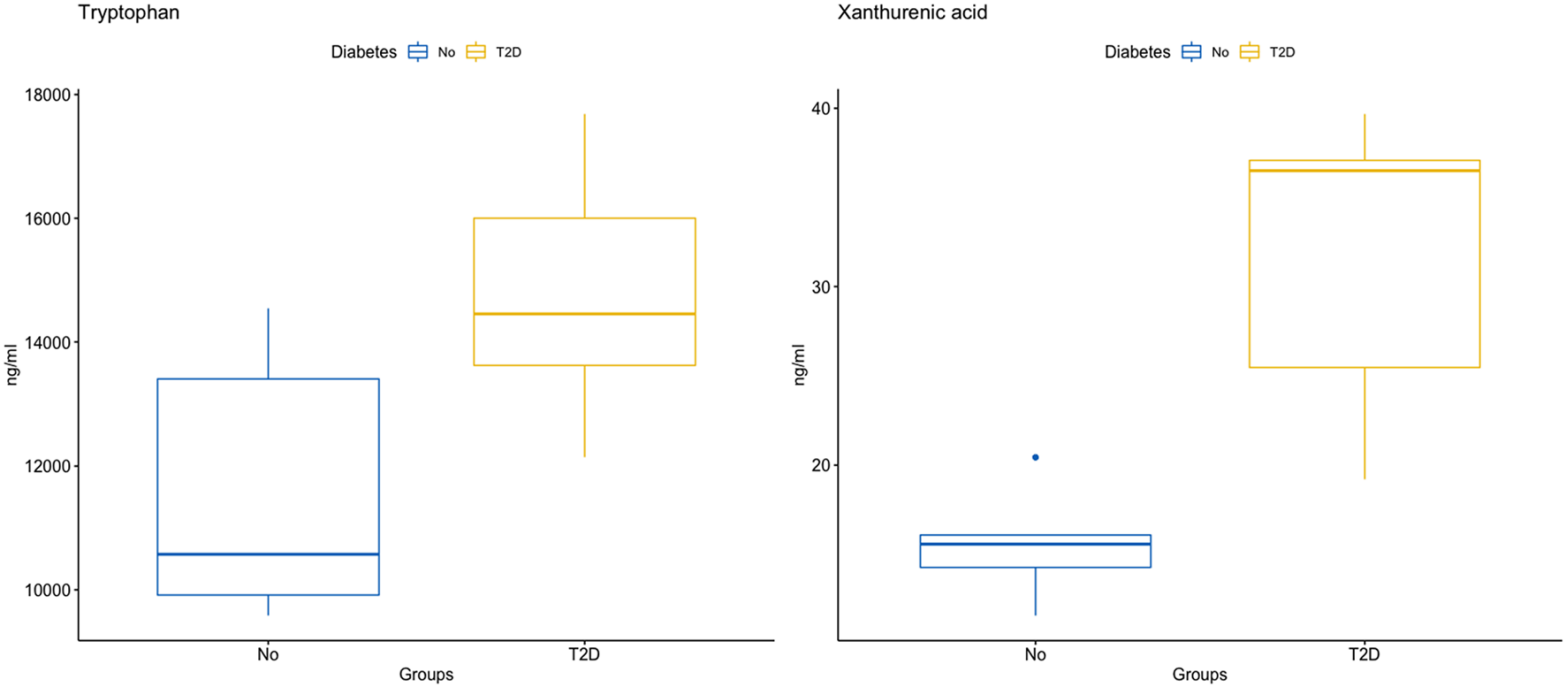
Baseline pre-operative concentrations of tryptophan and xanthurenic acid.

At pre-operative baseline, there was a significantly higher concentration of HbA1c in those participants with T2D, which was expected. In addition, serum tryptophan (p = 0.005) and xanthurenic acid (p = 0.015) concentrations were higher in those with T2D (Fig. 3), although after controlling for the FDR, BH q values for both metabolites were > 0.05 (q = 0.10 and q = 0.14 respectively).

Following surgical intervention, HbA1c concentrations were significantly reduced in both the T2D and Non-Diabetes (ND) groups, with a greater reduction observed in the T2D group. In the T2D group, significant reductions in the concertation of XA (p = 0.004, BH q = 0.030) and TRP (p = 0.003, BH q = 0.030) occurred (Fig. 2). No significant metabolite changes were observed in the ND group at the 3-month post-surgery timepoint (Table 5). The kynurenine/tryptophan ratio did not significantly change between pre- and post-surgery for either the T2D or the ND group.

**Table.5:** Effect of surgery by T2D subgroup on measured metabolites.

	T2D				ND			
	Pre Op	Post Op	p value	q value	Pre Op	Post Op	p value	q value
Picolinic acid	7.42 ± 3.82	5.83 ± 1.08	0.683	0.796	5.29 ± 1.56	6.68 ± 1.86	0.100	0.325
3 Hydroxyanthranilic acid	16.19 ± 7.56	14.3 ± 0.94	0.937	0.937	13.85 ± 4.39	NA ± NA	1.000	1.000
Quinolinic acid	66.57 ± 11.77	58.16 ± 25.84	0.113	0.318	69.18 ± 21.68	59.7 ± 19.11	0.353	0.675
Citrulline	3479.83 ± 1516.31	2950.59 ± 918.29	0.353	0.634	4261.41 ± 1451.63	3085.06 ± 872.6	0.063	0.301
Indole 3 acetic acid	318.45 ± 276.86	350.77 ± 238.45	0.579	0.737	271.94 ± 160.66	441.95 ± 375.87	0.529	0.820
Kynurenic Acid	8.22 ± 4.91	4.38 ± 3.79	0.011	0.054	7.54 ± 4.51	6.04 ± 4.27	0.579	0.820
5-Hydroxyindole acetic acid	9.98 ± 4.92	8.28 ± 2.32	0.571	0.737	7.78 ± 3.95	6.62 ± 2.02	0.631	0.820
Xanthurenic acid	31.59 ± 8.81	8.19 ± 5.33	0.004	**0.030**	15.58 ± 3.23	13.65 ± 8.73	0.364	0.675
Kynurenine	330.23 ± 49.48	282.51 ± 79.95	0.370	0.634	304.95 ± 85.57	262.71 ± 71.78	0.274	0.675
30H kynurenine	11 ± 3.98	7.33 ± 2.85	0.029	0.101	9.74 ± 3.63	6.78 ± 1.83	0.070	0.301
Neopterin	2.61 ± 1.72	3.03 ± 2.01	0.739	0.796	2.45 ± 0.84	2.37 ± 0.57	0.796	0.862
Tryptophan	14,794.55 ± 1848.93	9922.06 ± 3971.6	0.003	**0.030**	11,565.41 ± 2004.27	9822.78 ± 2557.61	0.052	0.301
Kynurenine/tryptophan ratio	0.023 ± 0.004	0.026 ± 0.006	0.408	0.634	0.027 ± 0.006	0.027 ± 0.006	0.762	0.862

q values were generated using the Benjamini and Hochberg method to control the false discovery rate (FDR).

Units of all of metabolites displayed—ng/ml.

KTR expressed as a ratio.

Significant values are indicated by bold type.

### Metabolite relationship with HbA1c

When evaluated with repeated measures correlation, tryptophan (r_rm_ = 0.798, p = 1.4e−5), kynurenic acid (r_rm_ = 0.629, p = 0.002) and xanthurenic acid (r_rm_ = 0.619, p = 0.006) were found to be positively associated with corresponding serum HbA1c concentrations at the time of sampling (Fig. 2). For r_rm_ values of all measured metabolites with HbA1c please refer to Supplementary 3.

### Metabolite relationship with BMI

**Figure 4:**
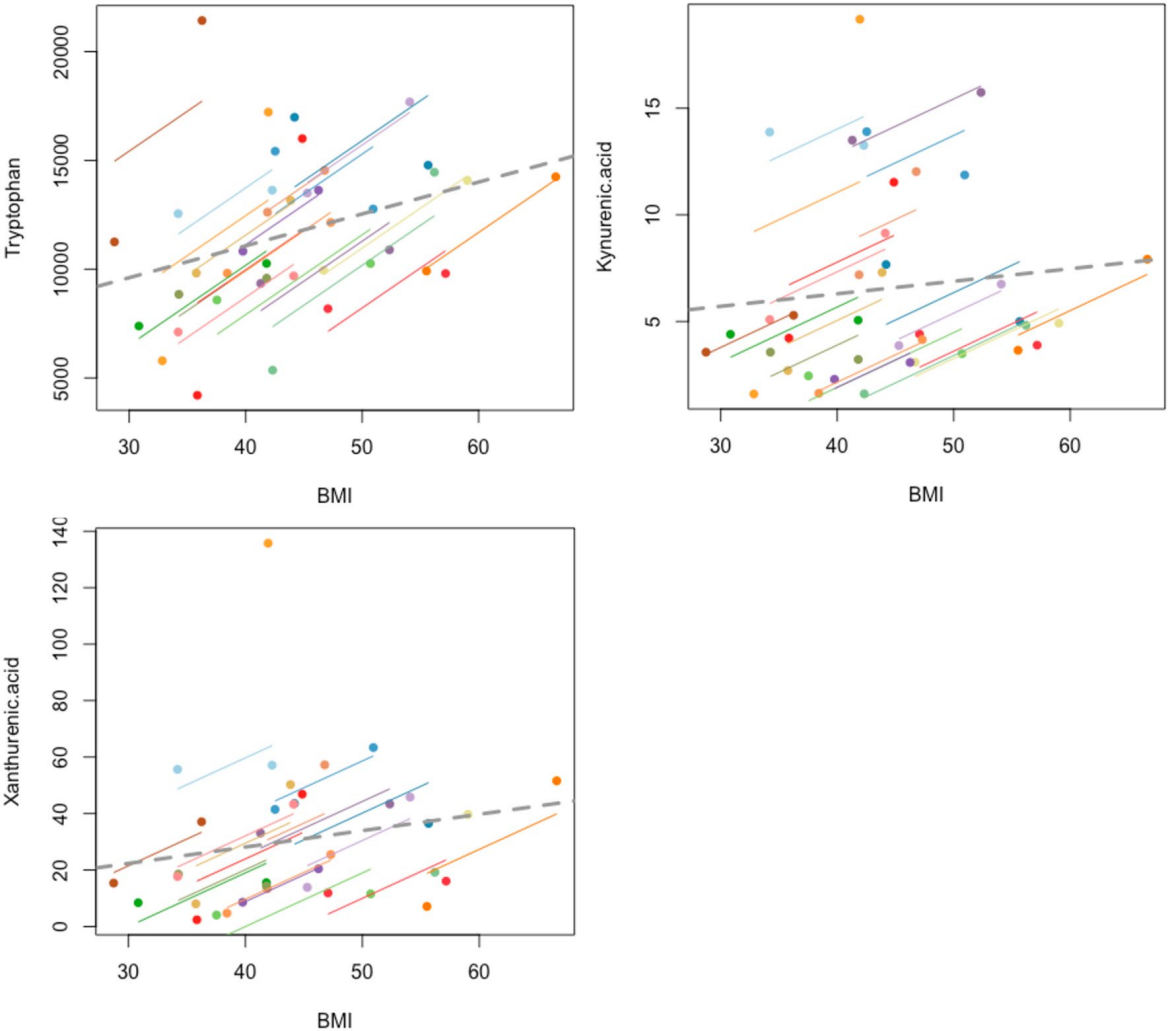
Repeated measure correlation plots of BMI with tryptophan, kynurenic acid and xanthurenic acid levels respectively. Metabolite units (ng/ml), BMI (kg/m^2^).

Similarly, when evaluated with repeated measures correlation, tryptophan (r_rm_ = 0.670, p = 9e−4), kynurenic acid (r_rm_ = 0.507, p = 0.018) and xanthurenic acid (r_rm_ = 0.693, p = 0.0014) were found to be positively associated with corresponding BMI measurement at the time of sampling (Fig. 4). For r_rm_ values of all measured metabolites with BMI please refer to Supplementary 4.

**Figure 5:**
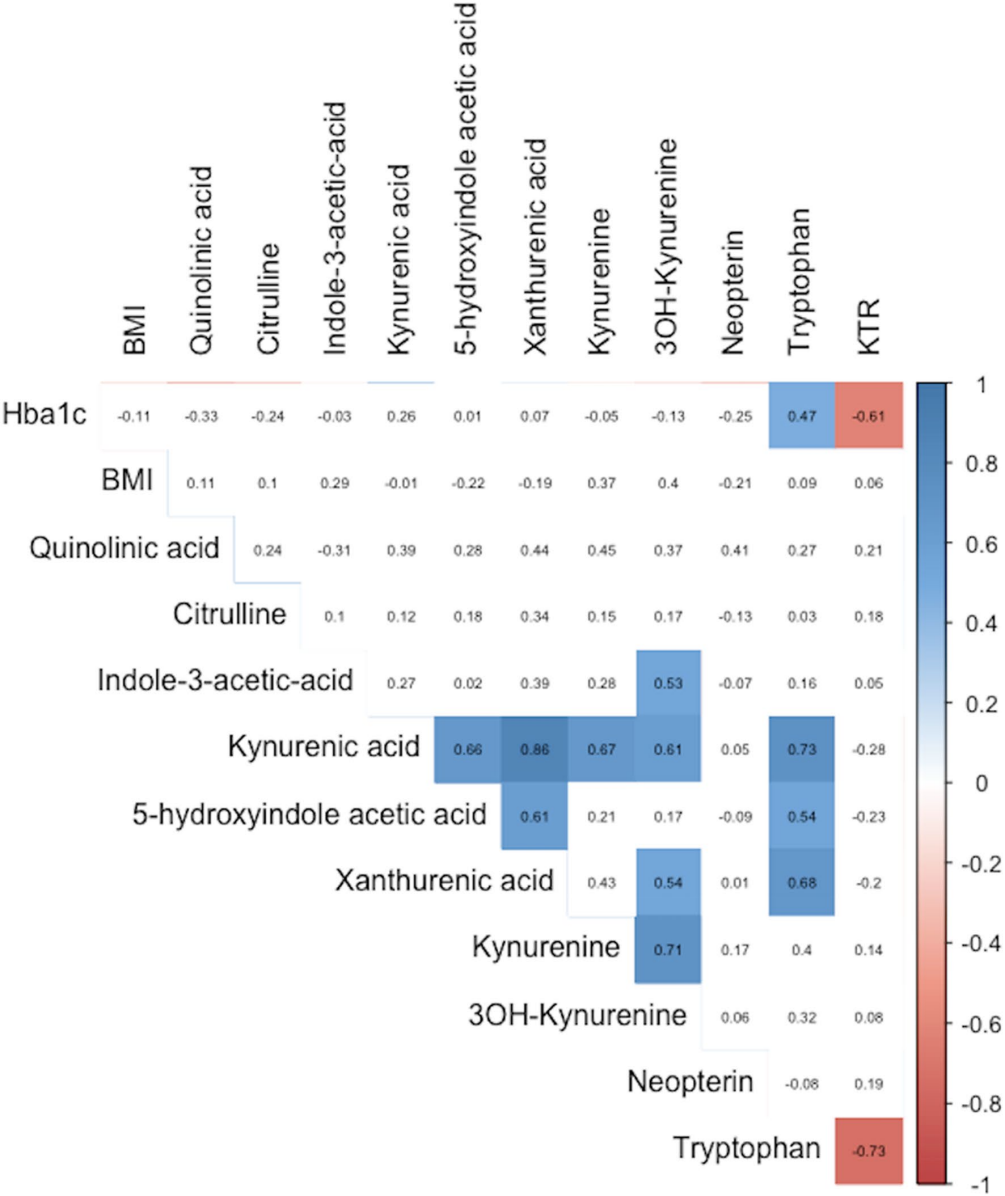
Heatmap displaying Spearman’s Rank correlation co-efficient between Δ of measured parameters and pathway metabolites. Coloured squares indicate significant results p value of < 0.05.

To further examine the effect of surgery on the KYN pathway, the correlation coefficient for the change (Δ) in concentration for each participant for each pathway metabolite with Δ BMI and ΔHbA1c was calculated (Fig. 5). Significant positive correlation was observed between ΔHba1c and ΔTryptophan (r = 0.57, p = 0.003) and significant negative correlation between ΔHba1c and ΔKTR (r = − 0.61, p = 0.004).

Changes in BMI were not found to be significantly correlated with any of the Δ of measured metabolites. Within the metabolic pathway, Δ Tryptophan showed a positive correlation with the downstream metabolites of Δ Kynurenic Acid (R = 0.73, p = 0.0002), Δ5-hydroxyindole acetic acid (R = 0.54 p = 0.013) and Δ Xanthurenic Acid (R = 0.68, p = 0.002).

## Discussion

Roux-En-Y gastric bypass (RYGB) but not vertical sleeve gastrectomy (VSG) led to significant decreases in serum tryptophan (TRP), kynurenic acid (KYNA) and xanthurenic acid (XA) levels. In those participants with T2D, surgery led to significant decreases in HbA1c and in serum TRP and XA levels. TRP, KYNA and XA levels were found to be strongly correlated with the corresponding serum HbA1c concentrations and BMI at the time of sampling when assessed using repeated measures correlation. Finally, Δ in serum TRP levels were found to be significantly and positively correlated with ΔHba1C and significantly and negatively correlated with Δ Kynurenine : Tryptophan Ratio (KTR). Δ BMI was not significantly correlated with any of the metabolites measured in this study.

Obesity induced inflammation and immunomodulation^30^ are believed to be one of the links between T2D and the tryptophan pathway^17^. Obesity is associated with increased gene expression of all key enzymes in the tryptophan-kynurenine pathway including IDO, KMO, kynureninase (KYNU) and kynurenine aminotransaminase (KAT) within adipose tissue and proinflammatory macrophages^31^. In particular, KMO activation may cause diversion of the pathway towards XA production. Our data support this with higher concentrations of XA reported in the pre-operative groups where BMI scores and obesity levels were greater, compared with the 3-month post-operative samples.

**Figure 6:**
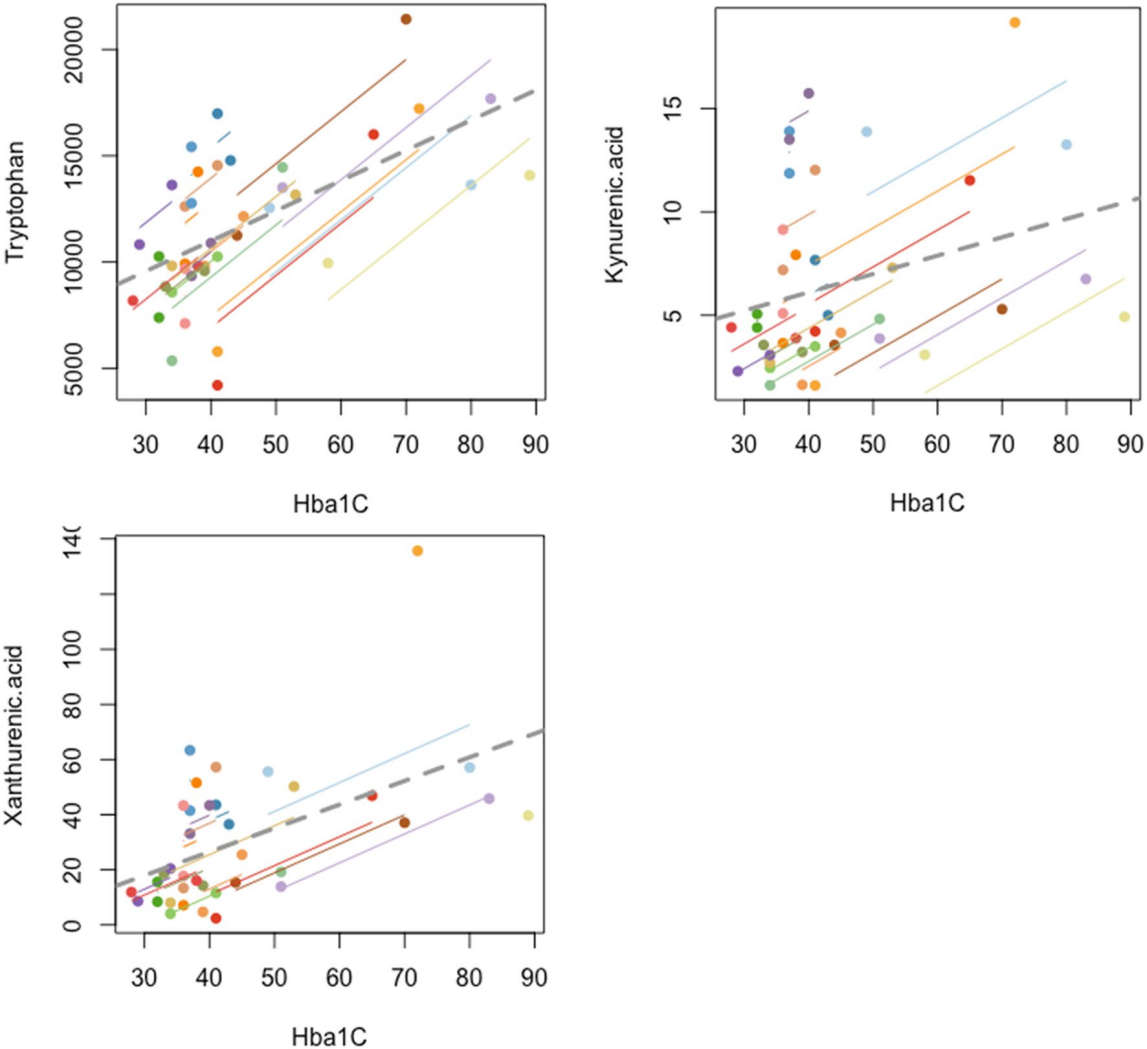
Repeated measure correlation plots of HbA1c with tryptophan, kynurenic acid and xanthurenic acid levels respectively.Metabolite units (ng/ml), HbA1c (mmol/mol).

KMO expression has been reported to be positively associated with HbA1c levels^31^ suggesting that there may be an association between its activity and clinical T2D. In addition, XA is considered to be diabetogenic with previous studies reporting raised levels of XA in the urine^32,33^ and serum^34^ in T2D. In relation to bariatric surgery, a reduction in XA has been reported with improved glucose control a year after surgery^35^. Our data are in agreement: we observed higher baseline XA concentrations in the T2D group compared to the ND group. In the T2D group, XA concentrations were dramatically reduced at the 3-month, post-operative timepoint, whilst concentrations in the ND group remained stable throughout the study. Interestingly, only surgical intervention with RYGB led to a significant reduction of XA at 3-months, whilst there were no significant changes in XA concentrations following the VSG procedure. XA also significantly correlated with HbA1c and BMI (Figs. 4, 6), again underscoring the association between XA and T2D. Although this observation does not demonstrate causality, the significant relationship supports the postulated contributory role of TRP-KYN pathway dysregulation in the pathogenesis of T2D.

Although the precise molecular mechanisms of XA role in T2D have not yet been elucidated, historical publications have proposed multiple mechanisms, including zinc chelation of XA leading to the formation of complexes that present identically to insulin antigens^36–38^, or the possibility that XA exhibits a direct toxic, and inhibitive effect on pancreatic islets^38–40^. The role of XA in T2D resolution post bariatric surgery warrants further investigation, as modulation of this pathway may be a target for future intervention in individuals with pre-surgical T2D.

In addition to KMO, the enzymes KYNU and KAT may also play a role in the relationship between the kynurenine pathway, obesity and T2D. For example, vitamin B6 is vital co-factor for both enzymes^41^, deficiency of which is also associated with obesity^42^ and chronic inflammation^41^. KYNU is more sensitive to vitamin B6 deficiency compared to KAT and thus KYNU activity is downregulated in instances of deficiency. This causes a pathway shift leading to increased production of XA and KYNA^43^, which is consistent with our data, where we observed higher concentrations of XA and KYNA in the pre-operative groups, where BMI and therefore levels of obesity were more extreme (Tables 3, 4). Furthermore, the reduction in BMI after surgery was also associated with reduced concentrations of these metabolites and improved glycaemic control, which potentially is due to the reduction in systemic inflammation.

The impact of bariatric surgery on serum KYN metabolites may also have implications beyond glycaemic control. Elevated kynurenic acid levels have been associated with an increased risk of acute coronary events in individuals with chronic heart disease^44^. In one study, tryptophan metabolite levels were shown to be positively correlated with severity of chronic kidney disease with a direct nephrotoxic effect hypothesised^45^. Improvement in renal function after bariatric surgery is now becoming more evident^46^, and regulation of tryptophan metabolites may also be partly responsible for improvement in such conditions.

There are also links between the TRP pathway and the central nervous system. Cytokine mediated inflammation has been implicated in dysregulation of the hypothalamus–pituitary–adrenal axis and can lead to depression^47^. Kynurenic acid and quinolinic acid are both of interest in neurogastroenterology. Both metabolites are neuroactive, with KYNA acting as a NMDA antagonist and QA considered to be a NMDA agonist^48^. These metabolites can influence neuronal activity in both the central and peripheral nervous systems, with the former being neuroprotective and the latter neurotoxic. From a bariatric surgery perspective, the key to relationship between tryptophan metabolism and the CNS may lie in the gut microbiome. The concept of the gut-brain axis is becoming more established^49^ and the gut microbiota are now thought to influence brain, mood and cognitive function. An example is found in patients with irritable bowel syndrome where TRP-KYN pathway metabolites kynurenic acid and serotonin levels measured in small bowel mucosa were found to be directly correlated with anxiety and depression scores. Furthermore, chronic low tryptophan states also appear to accelerate the conversion of serotonin to 5 hydroxyindoleacetic acid^50^. This growing body of research has led to the proposal of the hypothesis that changes in GI tract metabolism, associated with the TRP-KYN pathway, may influence psychological states^51^. As bariatric surgery reduces serum tryptophan, kynurenines and other macronutrient levels, it remains to be seen as to whether this effect may have an impact on the increased incidence of depression after bariatric surgery^52^ and warrants further investigation.

Finally, the actual bioavailability of tryptophan and therefore its downstream metabolites are reported to be directly influenced by the gut microbiome^49^. Gut microbes have been reported to metabolise tryptophan to indole species, which in turn play a mechanistic role in microbiota to host signalling^25,48^. Indoles, including IAA, that are produced from microbial metabolism of tryptophan are thought to act on the aryl hydrocarbon receptor (AhR) in humans and influence gut barrier function^53^. This relationship is becoming a focus of research in obesity^15^ and GI permeability post bariatric surgery^54^. In our data, there was no significant change in IAA concentrations in the pre- and post- surgery comparisons, indicating that gut-microbial metabolism of tryptophan was not an influencing contributing factor in the phenotypic changes observed. Further investigations into additional gut microbially derived indoles would be of benefit and would facilitate further exploration of the gut microbiome's influence on metabolism post bariatric surgery, however measurement of an additional metabolic pathway was beyond the scope of this study.

### Limitations

While longitudinal, this study only investigated the immediate metabolic changes 3 months after surgery. As a discovery study, the relatively small sample size limits the generalisability of the results and may decrease the power of the study. However, the widespread physiological and metabolic effects exerted by bariatric surgery means inter-individual variability is small compared to the overarching effects of the surgery^55^. The paired nature of this study also permitted us to measure and compare the differences in response between individuals as well as the surgical procedure. It will be important to evaluate the longer-term changes, ideally with further comparison groups of cohorts without obesity in order to validate the results.

There was an imbalance in the gender distribution of study participants (F:M, 15:5) which also needs to be considered: this however, was in keeping with the 3:1 (F:M) ratio of patients undergoing primary bariatric surgery in the United Kingdom^56^. Other factors that influence the TRP-KYN pathway such as vitamin B levels and the other derivatives of tryptophan such as the indole and its derivatives also merit investigation.

Finally, it should be considered that there is the potential that a reduction in overall nutrient intake post-surgery is driving the observations. A subset of 11 patients provided dietary information in the form of a recall online questionnaire (see Supplementary 5). General reductions in total calories consumed and all macronutrients were significant at 3-months post-operative. In particular, a reduction in average pre-operative protein intake of 80.6 g/day compared to 49.7 g/day post-operatively (p = 0.014) was reported. This is consistent with previous studies^57^ and could explain the reduction in tryptophan bioavailability. However, if dietary influence was the only factor at play in these observations, it would be expected that all metabolites in the pathway would be significantly reduced post-surgery, which was not the case, indicating the perturbations observed in the current study were associated with specific enzymatic steps of the pathway. A separate controlled study dedicated to investigating the effects of diet on the TRP-KYN pathway metabolites and its effects on T2D would be of interest as future work.

## Conclusion

Bariatric surgery, in particular RYGB reduces serum levels of tryptophan and its downstream kynurenine metabolites including kynurenic acid and xanthurenic acid. These metabolites are associated with T2D and its reduction after surgery correlates with a reduction in HbA1c levels and BMI. Whether this is a cause or consequence remains to be further established. The reduction of these metabolites may also have a wide range of beneficial and unintended effects beyond weight loss and glycaemic control.

## Supplementary Information

Supplementary Information.
