# Supplementary material for: The impact of bariatric surgery on serum tryptophan–kynurenine pathway metabolites
Source: Sci Rep. 2022 Jan 7;12:294. doi: 10.1038/s41598-021-03833-4 (PMC8741964; doi:10.1038/s41598-021-03833-4)

**Supplementary 1 Inclusion & Exclusion Criteria**

*Inclusion Criteria:*

Any patient referred to the Imperial Weight Centre (NHS) Tier 4 service team for consideration of bariatric surgery (RYGB / VSG) under NICE guidelines: Obesity (BMI>30kg/m^2^), Age ≥18, significant obesity associated comorbidities, failure of efforts at lifestyle modification and dieting, fitness for anaesthesia and procedure, willingness to comply with the trial protocol.

*Exclusion Criteria:*

Previous bariatric surgery and/or any previous major abdominal surgery including small or large bowel, liver, pancreatic, splenic resection surgery. Those who are currently pregnant or intention to become pregnant during trial period, lack of capacity to consent.

**Supplementary 2 Shapiro Wilk Test of Normality**

**[Supp Table 2: Shaprio Wilk Test of Measured Parameters and Metabolites]**


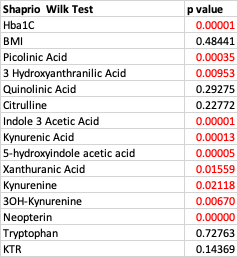


From the output, the p-value > 0.05 implies normality. In the case of this data set, the majority of variables are not normally distributed.

**Supplementary 3 Repeated Measures Correlation (HbA1c)**

**[Supp Table 3: Repeated Measures Correlation Co-efficient and p-value of metabolites measured against HbA1c]**

**Supplementary 4 Repeated Measures Correlation (BMI)**

**[Supp Table 4: Repeated Measures Correlation Co-efficient and p-value of metabolites measured against HbA1c]**

**Supplementary 5**

**[Supp Table 5: Dietary intake information from 11 patients. Each provided information on three occasions pre-operatively and post-operatively via an online recall questionnaire]**

*all listed data are means ± standard deviation (g/day) unless stated otherwise*

**[Supp Figure 5: Boxplots comparing Pre and Post-Operative Dietary intake information]**


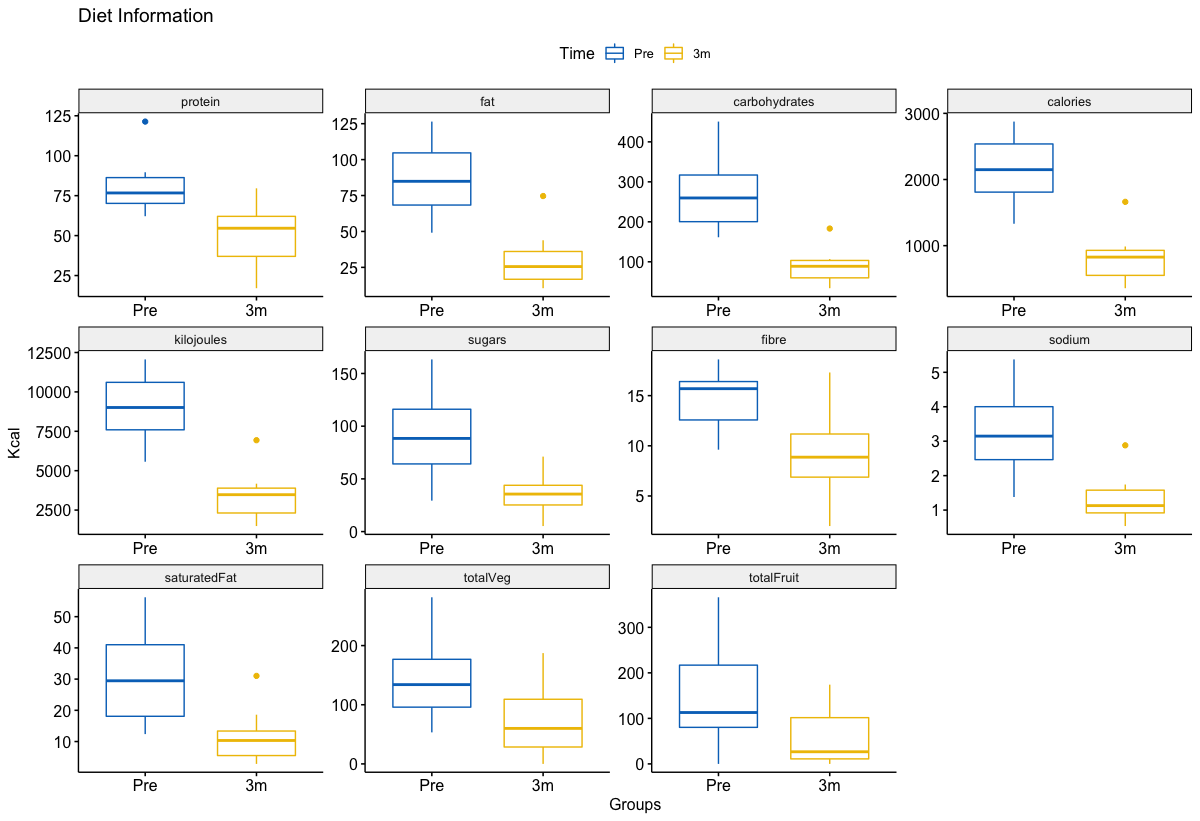

Supplement: Supplementary file 1 — Supplementary Information. [file 41598_2021_3833_MOESM1_ESM.docx]
